# Role of *Corynebacterium glutamicum sprA* Encoding a Serine Protease in *glxR*-Mediated Global Gene Regulation

**DOI:** 10.1371/journal.pone.0093587

**Published:** 2014-04-01

**Authors:** Eun-Ji Hong, Joon-Song Park, Younhee Kim, Heung-Shick Lee

**Affiliations:** 1 Department of Biotechnology and Bioinformatics, Korea University, Sejong-ro, Sejong-si, Korea; 2 Department of Oriental Medicine, Semyung University, Checheon, Chungbuk, Korea; University of Strathclyde, United Kingdom

## Abstract

The global regulator *glxR* of *Corynebacterium glutamicum* is involved in many cellular activities. Considering its role, the GlxR protein likely interacts with other proteins to obtain, maintain, and control its activity. To isolate proteins interacting with GlxR, we used a two-hybrid system with GlxR as the bait. Subsequently, the partner, a subtilisin-like serine protease, was isolated from a *C. glutamicum* genomic library. Unlike *glxR*, which showed constitutive expression, the expression of *sprA*, encoding a serine protease, was maximal in the log phase. Purified His_6_-SprA protein underwent self-proteolysis and proteolyzed purified GlxR. The proteolytic action of SprA on GlxR was not observed in the presence of cyclic adenosine monophosphate, which modulates GlxR activity. The *C. glutamicum sprA* deletion mutant (Δ*sprA*) and *sprA*-overexpressing (P_180_-*sprA*) strains showed reduced growth. The activity of isocitrate dehydrogenase (a tricarboxylic acid cycle enzyme) in these strains decreased to 30–50% of that in the wild-type strain. In the P_180_-*sprA* strain, proteins involved in diverse cellular functions such as energy and carbon metabolism (NCgl2809), nitrogen metabolism (NCgl0049), methylation reactions (NCgl0719), and peptidoglycan biosynthesis (NCgl1267), as well as stress, starvation, and survival (NCgl0938) were affected and showed decreased transcription. Taken together, these data suggest that SprA, as a serine protease, performs a novel regulatory role not only in *glxR*-mediated gene expression but also in other areas of cell physiology. In addition, the tight control of SprA and GlxR availability may indicate their importance in global gene regulation.

## Introduction

*Corynebacterium glutamicum* is a non-pathogenic, Gram-positive organism that belongs to the order *Actinomycetales*. Based on its metabolic capability, this organism has been widely used in the industrial production of nucleotides and amino acids [1]. Therefore, the catabolic and anabolic pathways associated with amino acid metabolism have attracted scientific attention and have been extensively analyzed. Moreover, the availability of genome sequence data [2]–[4] has significantly increased the understanding of gene expression and regulation on a global scale [5].

The global regulator *glxR* of *C. glutamicum* was initially identified through its capability to repress glyoxylate bypass genes [6]. The bypass, mediated by isocitrate lyase (ICL) and malate synthase (MS), is not required by cells grown with glucose as the sole carbon source, and therefore the genes mediating the bypass are repressed by *glxR*. However, when cells are provided with two-carbon compounds, such as acetate, the bypass plays a critical role because it conserves carbon by bypassing the CO_2_-generating steps of the tricarboxylic acid cycle. ICL (encoded by *aceA*) catalyzes the conversion of the tricarboxylic acid cycle intermediate isocitrate into glyoxylate and succinate, and MS (encoded by *aceB*) catalyzes the subsequent condensation of glyoxylate with acetyl-coenzyme A to produce malate. Furthermore, transcriptional regulators such as RamA and RamB are also involved in the regulation of *aceA* and *aceB* expression [7]–[9].

GlxR is a cyclic adenosine monophosphate (cAMP)-binding transcriptional regulator [10] that belongs to the cAMP receptor protein/fumarate and nitrate reduction regulator (CRP/FNR) family of proteins and shows homology to the global regulator CRP from *Escherichia coli* [6]. In addition to its role in acetate metabolism, *glxR* is involved in many other cellular activities, such as carbon metabolism [6], [11]–[14], energy metabolism [15], lipid metabolism [16], anaerobic nitrate respiration [17], inorganic phosphate uptake [18], and cell resuscitation [19]. Further, Kohl et al [20] analyzed the whole genome using *in silico* tools in combination with *in vitro* analysis and predicted that the GlxR regulon comprises many genes (96 genes in 53 transcription units) presumed to be involved in diverse cellular activities such as carbohydrate metabolism, aromatic compound degradation, glutamate uptake and nitrogen assimilation, fatty acid biosynthesis, stress response, and resuscitation. The global function of GlxR has recently been confirmed and expanded by Jungwirth et al [21] and Toyoda et al [22], who analyzed GlxR-bound target DNA sequences isolated from cells via immunoprecipitation. A total of 14% of *C. glutamicum* genes may be under the direct transcriptional control of GlxR [23].

Despite the importance of GlxR as a global regulator, many questions regarding its mechanism and the control of its action remain largely unanswered, partly owing to the essential role of *glxR* in cell physiology. For example, in earlier studies, *glxR* deletion mutants could not be isolated [6], [13] or grew too slowly for study when isolated [24]. However, Park et al [25] were able to isolate a *glxR* deletion mutant; they demonstrated that the mutant strain had a severe growth defect and that *glxR* repressed glyoxylate bypass genes. Incidentally, GlxR functions as a dual regulator that can be a repressor or an activator depending on its target genes. The position of its consensus binding site TGTGA-N_2_-TA-N_2_-TCACA [20] relative to the transcriptional start site also supports its dual role. In all the cases studied, the *in vitro* binding of GlxR to its target binding sites depended on the presence of cAMP. Unlike the findings in *E. coli*, intracellular cAMP levels in *C. glutamicum* are reported to be elevated during growth on glucose and low during growth on acetate [6].

Although only a small degree of information is available about the regulation of global regulatory proteins, it is clear they must also be subject to a regulatory process. The autoregulation of transcription occurs for many regulatory genes [5]. Activity regulation with effector molecules is also commonly seen with many regulatory proteins [10]. In addition, proteolytic control of regulators to maintain their appropriate intracellular concentrations is also seen in the case of some proteins [26]. Considering the importance of global regulators, several regulatory mechanisms are thought to be present for these genes and proteins. This type of regulation may also occur for GlxR in order to ensure its optimum performance under the physiological conditions of the cell. GlxR is very likely to interact with other proteins to obtain, maintain, or control its activity. For this reason, we aimed to isolate proteins interacting with GlxR by implementing a two-hybrid screening system. We used GlxR as bait and screened the *C. glutamicum* genome. Herein, we report the identification of a subtilisin-like serine protease encoded by *sprA* as an interacting partner for GlxR. Given the physiological and biochemical data, we propose a role for *sprA* in *glxR*-mediated global gene expression.

## Materials and Methods

### Bacterial Strains and Growth Conditions

*C. glutamicum* AS019E12 [27] was used to construct HL1385, which harbored a Δ*sprA* mutation. *C. glutamicum* HL1516 and HL1389 harbored the *sprA*-complementing plasmid pSL535 and *sprA*-overexpressing plasmid pSL509, respectively. *E. coli* DH10B (Invitrogen) was used for the construction and propagation of plasmids. *E. coli* BL21-CodonPlus (DE3)-RIL (Stratagene) and *E. coli* DH5αF′ (Bethesda Research Laboratories) were used for the expression of histidine-tagged SprA (His_6_-SprA) and maltose-binding protein (MBP)-fused GlxR (MBP-GlxR), respectively. *E. coli* and *C. glutamicum* strains were cultured in Luria-Bertani broth at 37°C and MB medium at 30°C, respectively [27]. MCGC minimal media for *C. glutamicum* were prepared as described previously [28]. Glucose and acetate were added as carbon sources to the MCGC minimal medium at 1% and 2% (w/v), respectively. Selective and nonselective broths (BacterioMatch II Two-Hybrid System, Agilent Technologies) for *E. coli* XL1-Blue MRF′ kan were prepared as described [29]. Antibiotics were added at the following concentrations (μg mL^−1^): 50 ampicillin, 5 tetracycline, 20 chloramphenicol, and 25 kanamycin.

### DNA and RNA Analyses

Plasmids were introduced into *C. glutamicum* cells through electroporation [30]. Total RNA isolation for *C. glutamicum* was performed using the FastPrep24 system (MP Biomedicals). Polymerase chain reaction (PCR) was performed as previously described [6]. cDNA conversion and real-time quantitative PCR (RT-qPCR) were also performed as previously described [29]. CFX96 Real-Time PCR Detection System (Bio-Rad) was used for gene expression analysis. Standard curves, expression normalization, and standard error values were obtained using CFX Manager software ver. 1.5 (Bio-Rad), which employs the ΔΔC_t_ method, and16S rRNA was used for normalization. RT-qPCR products were verified by melting curve and peak analyses. The following primers were used: *glxR*, 5′-CACCGAAGTTCATGCAGCAACCAT-3′ and 5′-TTAGCCAGCTGCAGAAGGGTCTTA-3′; NCgl0550, 5′-AGCTTGGCCGCTCTGTTTATGTTG-3′ and 5′-AGTTGCTAGTTGGTGAGCTTGGGA-3′; NCgl0719, 5′-AGAGCCAGCTGGTGTTCCAGTATT-3′ and 5′-TCGAGGATCATGTTTGGCAGCTCA-3′; NCgl0938, 5′-AGCCATCGTCCCAATTTCAAAGGC-3′ and 5′-TGATCTCATCGGTGACAACTCGCA-3′; NCgl2809, 5′-TCACCTGCGAAAGCTCCGATAACA-3′ and 5′-ATTACGCGCATCTTCGCACCAAAG-3′; and 16S ribosomal RNA, 5′-CGGCCTATCAGCTTGTTGGT-3′ and 5′-TGGGCCGTGTCTCAGTCC-3′.

### Plasmid and Strain Construction

Plasmid pSL500 harboring *glxR* cloned into the pBT vector (Agilent Technology) was constructed by introducing the *Eco*RI- and *Bam*HI-digested fragment, which was amplified from the *C. glutamicum* chromosome with primers 5′-CCGGAATTCAGTGGAAGGTGTACAG-3′ and 5′-CCGGGATCCTGGTGATTATCG-3′, into the vector. Plasmid pSL501 (pTRG-NCgl0550), harboring *sprA* (i.e. NCgl0550), was constructed as follows. The chromosomal gene was amplified with the primers 5′-CGCGGATCCTTGGATTCGGTGG-3′ and 5′-GGACTAGTGGATTCTGAACGGT-3′ and cloned into the pTRG vector (Agilent Technology). The plasmids pSL502 (pTRG-NCgl2548), pSL503 (pTRG-NCgl1430), pSL504 (pTRG-NCgl2510), and pSL505 (pTRG-NCgl1535) were constructed using the procedure described above but with the following primers: pTRG-NCgl2548, 5′-CCGGAATCCCCACACTGACCAT-3′ and 5′-CCGCTCGAGCCAGATCTTGTCAG-3′; pTRG-NCgl1430, 5′-CCGGAATTCTAGGGTTGGAGACTATG-3′ and 5′-CCGCTCGAGGGAGAAGAATGCTCA-3′; pTRG-NCgl2510, 5′- CGCGGATCCCAGATAGGCTTGTC-3′ and 5′-GGACTAGTCCCCACGAACCGAAAC-3′; and pTRG-NCgl1535, 5′-CCGGAATTCTCGCGCAGTAAATGG-3′ and 5′-GGACTAGTGCTCCTCGACAATACCT-3′. The pSL510 plasmid expressing the His_6_-SprA fusion protein was constructed via the amplification of *sprA* using the primers 5′-CCCAAGCTTCGGTGGATAAGATG-3′ and 5′-CCGCTCGAGGTGTAGTTGGTTTGG-3′, digesting the fragment with *Hin*dIII and *Xho*I, and ligating the 1.3-kb PCR product into the *Hin*dIII- and *Xho*I-digested pET28a vector. The pSL509 plasmid (P_180_-*sprA*), used to overexpress the *sprA* gene, was constructed via amplification of the *sprA* gene using the primers 5′-CTGCAGGGATTCGGTGGATA-3′ and 5′-CTGCAGGGTGCCTCATAGAT-3′ and ligating the amplified DNA into the *Pst*I site of pSL360 [31]. Plasmid pSL360 is an expression vector harboring the P_180_ promoter that overexpresses the fused gene. Overexpression of the *sprA* gene was verified by measuring the messenger RNA (mRNA) levels of *sprA* using RT-qPCR. Plasmid pSL535, harboring *sprA* with its own promoter, was constructed as follows. The 1.3-kb *sprA* gene was amplified with the primers sprAorfF2 5′-TTTTGGGGGCGGTGGATAAGATGCG-3′ and sprAorfR2 5′- CGCGGATCCGATGGGATTCTGAAC-3′. The 310 bp promoter region was amplified with the primers sprApF1 5′-CCGCTCGAGGTACCGCTTGTGGCA-3′ and sprApR1 5′- TTATCCACCGCCCCCAAAATTGTCCCCC-3′. The amplified 1.3-kb and 310-bp fragments were ligated and inserted into the pMT1 vector [27] previously digested with *Bam*HI and *Xho*I.

The *C. glutamicum* Δ*sprA* mutant strain was constructed according to a method described by Schäfer et al [32]. Briefly, a DNA fragment was prepared from the *C. glutamicum* genome using crossover PCR with the following primers: F1, 5′-CGCGGATCCTTGGATCGGTGG-3′; R1, 5′-CCTGAATGTGCCGTCGCAATCGATAAGTTC-3′; F2, 5′-ATTGCGACGGCACATTCAGGCTGGTCTATC-3′; R2, 5′-CGCGGATCCGGTGCCTCATAGAT-3′. The amplified fragment was cloned into the pGEM-T Easy vector (Promega). The *Bam*HI fragment was then isolated and inserted into *Bam*HI-digested pK19mobsacB [32]. Subsequent procedures were conducted as previously described [30], [33], and the chromosomal deletion of *sprA* in *C. glutamicum* HL1385 was verified by PCR with the primers F1 and R2.

### Two-hybrid System

A *C. glutamicum* genomic library was prepared as previously described [29]. The BacterioMatch II Two-Hybrid system (Agilent Technology) was used according to manufacturer instructions. Briefly, the two plasmids, pBT and pTRG, containing the “bait” and “target” genes, respectively, were used to transform *E. coli* simultaneously. Protein–protein interactions were screened based on the expression of *his3* and *aadA*, which confer histidine prototrophy (His^+^) and streptomycin resistance (Str^+^), respectively. For screening, 50 ng of each pSL500 (i.e., pBT-*glxR*) and target library DNA were introduced into reporter cells and spread onto selective media (His^−^ and Str^+^). Colonies were isolated and the plasmids in the growing cells were analyzed.

### Protein Purification and Proteolytic Assays

MBP-GlxR was purified as previously described [6]. His_6_-SprA was overexpressed, isolated, and refolded on a HisTrap FF column (GE Healthcare) as described in the Recombinant Protein Purification Handbook (GE Healthcare). Briefly, we grew *E. coli* BL21-CodonPlus (DE3)-RIL cells harboring plasmid pSL510 in 300 mL Luria-Bertani broth to an optical density of 0.5 at 600 nm, added isopropylthio-β-galactoside to a final concentration of 0.3 mM, and harvested the cells after an additional 2 h of incubation. Cells were resuspended in 10 mL buffer (20 mM Tris-HCl, 100 mM NaCl, pH 8.0) and disrupted by sonication. Inclusion bodies were collected via centrifugation at 9,500 × *g* for 1 h, washed twice with buffer (2% Triton X-100, 10 mM Tris-HCl, 100 mM NaCl, 2 M urea, pH 8.0), and incubated at room temperature for 4 h in 10 mL denaturation buffer (8 M urea, 20 mM Tris-HCl, 100 mM NaCl, 10 mM imidazole, pH 8.0) for denaturation. Solubilized proteins were recovered by centrifugation at 9,500 × *g* for 1 h, followed by passage of the supernatant through a syringe filter (0.45 μm). Proteins were loaded onto a Ni^+^ column (1 mL HisTrap His-FF, GE Healthcare), which was subsequently washed with 10 mL denaturation buffer at a flow rate of 0.1 mL min^−1^. Refolding of His_6_-SprA was induced by slowly (0.1 mL min^−1^) replacing the denaturation buffer with 20 mL refolding buffer (20 mM Tris-HCl, 100 mM NaCl, 10 mM imidazole, 5 mM dithiothreitol, pH 8.0) through the application of a linear gradient. The proteins were then eluted with 5 mL elution buffer (20 mM Tris-HCl, 100 mM NaCl, 1 M imidazole, pH 8.0) at a flow rate of 1 mL min^−1^. Proteins were concentrated via ultrafiltration (Amicon Ultra Centrifugal Filter Devices, Millipore), and the buffer was replaced with assay buffer (40 mM potassium phosphate, pH 8.0).

Proteolytic assays were conducted in a total volume of 25 μL at 30°C by incubating proteins for 0–24 h. The assay buffer was composed of 40 mM potassium phosphate (pH 8.0). Subsequently, the assay mixtures were analyzed by sodium dodecyl sulfate polyacrylamide gel electrophoresis (SDS-PAGE). The proteins in the assay mixtures were as follows (pmol): 200 MBP-GlxR, 400 GlxR, 630 His_6_-SprA, and 111 bovine serum albumin (BSA).

### Two-dimensional (2D)-PAGE and Enzyme Assays

2D-PAGE was conducted as previously described [30], [34]. Before PAGE, protein extracts were solubilized in a rehydration buffer containing 9 M urea, 2 M thiourea, 4% (wt/vol) 3-[(3-cholamidopropyl)-dimethylammonio]-1-propanesulfonate, 0.4 M Tris, 0.1 M (wt/vol) dithiothreitol, and 0.5% (vol/vol) IPG buffer (GE Healthcare) for 1 h. After rehydration, isoelectric focusing and second-dimension analyses were performed as described earlier [34]. Protein spots were visualized with Coomassie brilliant blue staining, and peptide analysis was performed by a commercial service (Proteinworks) via electrospray ionization mass spectroscopy.

Cell lysates of *C. glutamicum* cells were prepared as described previously [6] and enzymatic activities of MS, ICL, and isocitrate dehydrogenase (ICDH) were determined as previously described [35].

## Results

### Isolation of Proteins Interacting with GlxR

**Figure 1:**
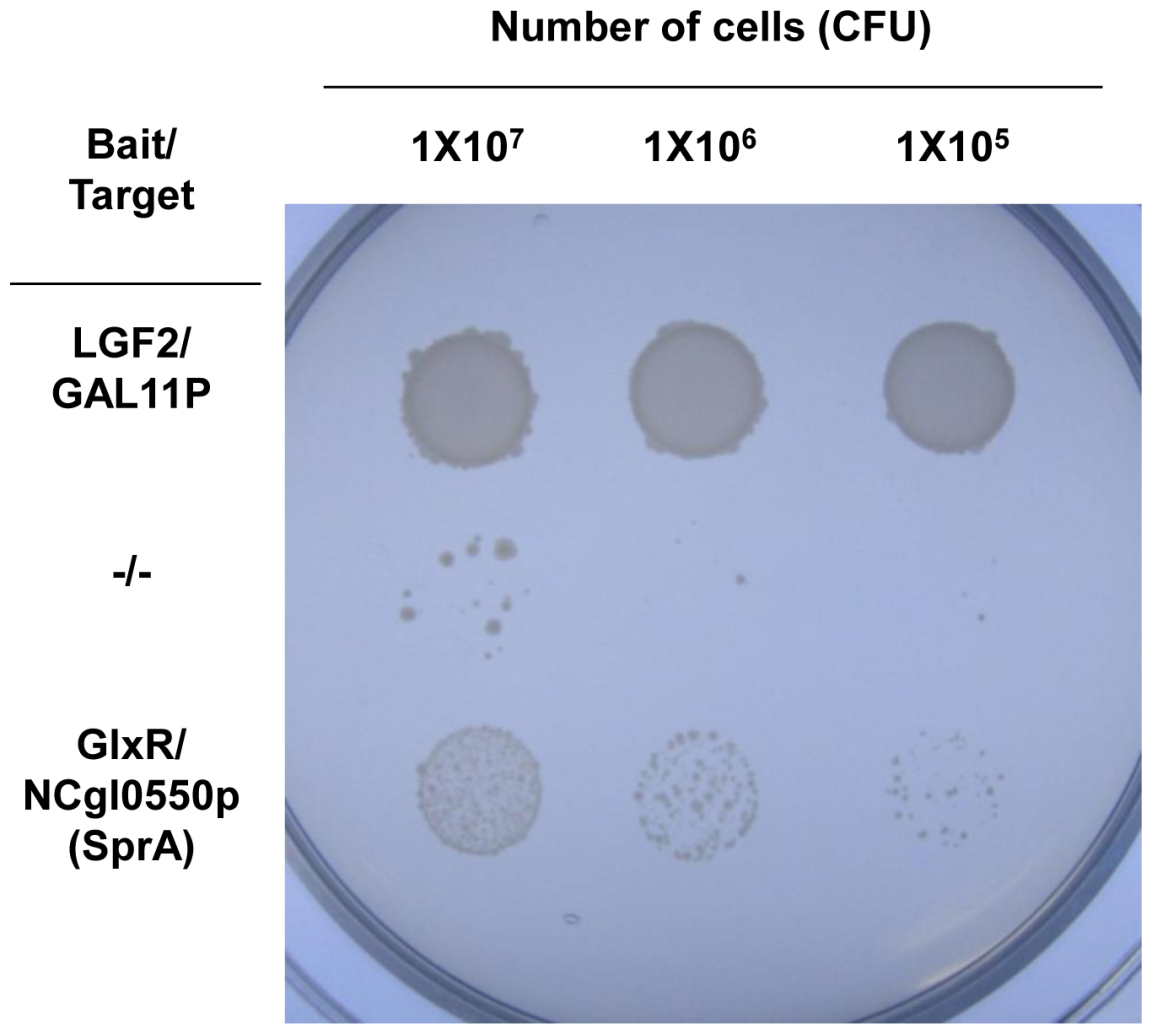
Interaction of GlxR with SprA in the two-hybrid system. The interaction was assayed by monitoring the growth of cells harboring bait and target proteins. Positive control cells harbored well-established interacting proteins such as LGF2 as bait and Gal11p as the target. Negative control cells harbored empty pBT (bait) and pTRG (target) vectors. NCgl0550p, NCgl0550-encoded protein; and CFU, colony-forming units.

To isolate protein(s) interacting with GlxR, we used a two-hybrid system with pSL500, which expresses GlxR, as the bait vector. Protein-protein interactions were screened based on the expression of *his3* and *aadA*, which confer His^+^ and Str^+^, respectively. Introduction of the *C. glutamicum* genomic library into *E. coli* reporter cells harboring the bait vector allowed us to isolate five target clones exhibiting the His^+^ and Str^+^ phenotypes. Subsequently, the plasmids were isolated, and the DNA fragments in the “target” vector were sequenced. Two of the clones contained gene fragments that expressed peptide sequences, which turned out to be subtilisin-like serine protease (NCgl0550) and Xaa-proline peptidase (NCgl1430; Table 1). To verify the interaction of the proteins with GlxR, we cloned the full-length open reading frames of NCgl0550 and NCgl1430 into the target vector, introduced them into reporter cells carrying the *glxR*-carrying bait vector, and monitored their growth on selection media. Only the cells carrying the NCgl0550 gene showed growth on the medium (Figure 1). To quantify the protein-protein interaction, we measured the transcriptional level of the reporter gene *his3* by RT-qPCR and determined it to be 11.3% compared with that of the positive control cells, which was set at 100%. The value observed in cells harboring empty vectors was 3.4%. Considering that the screening conditions may not have been physiologically ideal for inducing protein interactions (see Discussion), we tentatively concluded that GlxR specifically interacts with the NCgl0550-encoded protein. We then analyzed the gene further to see if the protein product interacted with GlxR and whether this interaction had physiological significance.

**Table 1:** Screened proteins and peptide sequences that interact with GlxR.

Screened sequence	Open reading frame with similarity	Annotated function	Frame^1^	ID^2^
GSTVYPAHSDTVLSVSARFDSHTLAEYSMPGNQQILSAPSHIQAGLSPRGDGFASHMIRGRKNSV	NCgl0550 (*sprA*)	Subtilisin-like serine protease	In frame	100
GSAENGANPHHGFSDRVLRNGDIVVVDIGGTFGPGYHSDCTRTYIVGGNPDDADPRPQEFSLSWRSSN	NCgl1430	Xaa-Pro aminopeptidase	In frame	93
GSSSVPLPWCAPTFSGLPLKVQKHARRLAHHHQKQIRGSRQ	NCgl1535	Pyrimidine reductase	Out of frame	56
GSTLPPPDDHLVCWGQQRP	NCgl2548	Hypothetical protein	Out of frame	46
GSHYLPSLLSVLCSHGKTPKSSSWHANPGLQPFST	NCgl2510	Pyridoxal-5′-phosphate -dependent aminotransferase	Out of frame	28

1 Reading frame of the cloned gene that produced the screened sequences.

2 Identity (percent of matched amino acid sequence).

### Analysis of NCgl0550

The open reading frame of NCgl0550 was 1,245 bps long and encoded a 43,143-Da protein composed of 414 amino acids. The NCgl0550-encoded protein had conserved features found in subtilisin-like serine proteases (InterProScan program, http://www.ebi.ac.uk/Tools/pfa/iprscan), such as hydrophobic domains in the N-terminus and near the C-terminus and a catalytic triad characterized by the conserved amino acids Asp, His, and Ser [36], [37]. The catalytic triad within conserved sequences D90, H121, and S332 is common among bacterial serine proteases of the subtilisin family. In addition, the encoded protein exhibited typical subtilisin family domain architecture, which is most common among subtilisin-like serine proteases and is associated with a proposed biological function of proteolysis [38]. In addition, a putative signal peptide cleavage site (between the 23rd and 24th amino acids) was also found in the N-terminal region of the protein, suggesting that the protein could be secreted or membrane anchored. The encoded protein showed approximately 35% identity with the mycosins (encoded by *mycP1-5*), which are subtilisin-like serine proteases, of closely related *Mycobacterium tuberculosis* H37Rv, whereas the five serine proteases of the organism have been reported to show 36–47% identity with each other [39]. The encoded protein also showed 32% identity with the subtilisin-like serine protease (YaB, encoded by *ale*) of *Bacillus subtilis* [40]. Homologies with other subtilisin-like serine proteases were generally low (data not shown). Based on the homology with serine proteases, NCgl0550 was designated as *sprA* (serine protease A).

**Figure 2:**
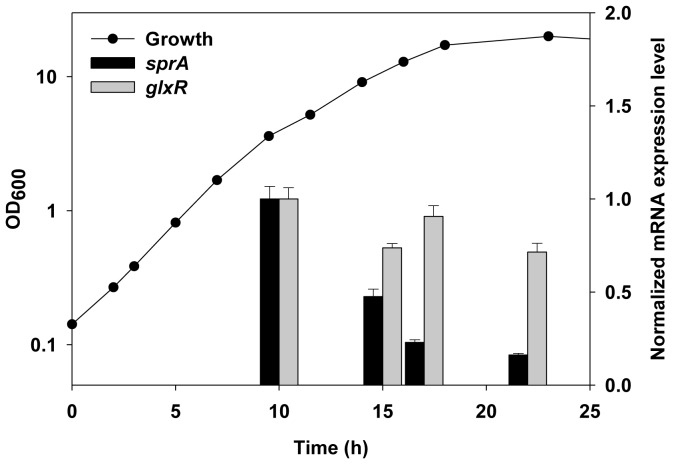
Expression of *glxR* and *sprA* during growth. Cells were grown in glucose MCGC media, and mRNA levels were measured by RT-qPCR as described in the Materials and Methods. The data represent three independent experiments. Filled circles indicate the growth of *C. glutamicum*. Bars indicate mRNA levels of *glxR* (grey) and *sprA* (black), respectively. OD, optical density.

To study the role of *sprA* in *glxR*-mediated regulation, we first used RT-qPCR to analyze the expression profile of *sprA* during growth. The expression of *glxR* showed only marginal differences, whereas that of the *sprA* gene was maximal in the log phase and gradually decreased as the cells entered the stationary phase, suggesting that it plays a role in the active growth phase (Figure 2).

**Figure 3:**
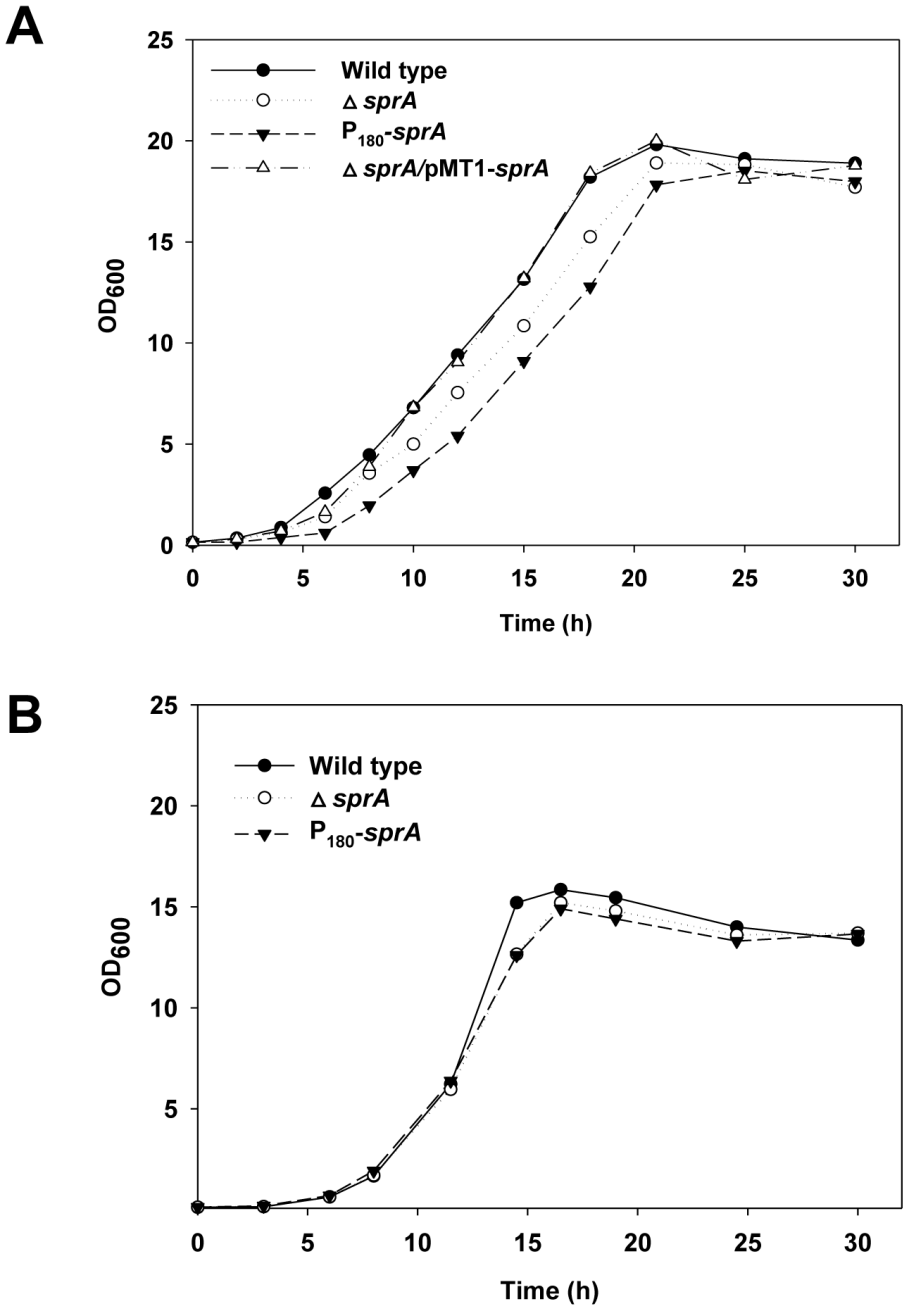
Growth characteristics of *C. glutamicum* wild type and mutants. Cells were grown on glucose (A) or acetate (B) MCGC minimal media. The data represent three independent experiments. Symbols: •, wild-type *C. glutamicum*; ○, *C. glutamicum* Δ*sprA*; ▾, *C. glutamicum* harboring pSL509 (P_180_-*sprA*); ▵, *C. glutamicum* harboring pSL535 (pMT1-*sprA*).

To determine the importance of *sprA*, we constructed a *C. glutamicum sprA* deletion mutant (Δ*sprA*) and a *sprA*-overexpressing strain (P_180_-*sprA*) and monitored their growth properties on minimal media. Internal deletion of a 494-bp fragment in *sprA* was verified by PCR (data not shown). The identity of the deleted gene was verified by complementing the Δ*sprA* strain with the plasmid pSL535 (i.e., pMT1-*sprA*), which harbors the *sprA* gene with its own promoter; the complemented strain showed wild type-like growth (Figure 3A). Promoter P_180_ is known to overexpress the fused gene irrespective of the growth phase [31]. Overexpression of *sprA* (approximately 15-fold relative to that of the wild-type strain) was confirmed by RT-qPCR. As shown in Figure 3A, when grown on glucose minimal media, the Δ*sprA* and P_180_-*sprA* strains exhibited reduced growth in the log phase relative to that of the wild-type strain, requiring longer incubation time to reach the stationary phase. In accordance with the expression data (see Figure 2), deletion of the gene did not significantly affect cell growth in the stationary phase. The differences in growth were further minimized when the cells were grown on acetate minimal medium (Figure 3B). Collectively, these data suggest a role for *sprA* in central metabolism during growth.

### *In vitro* Proteolysis of GlxR by SprA

**Figure 4:**
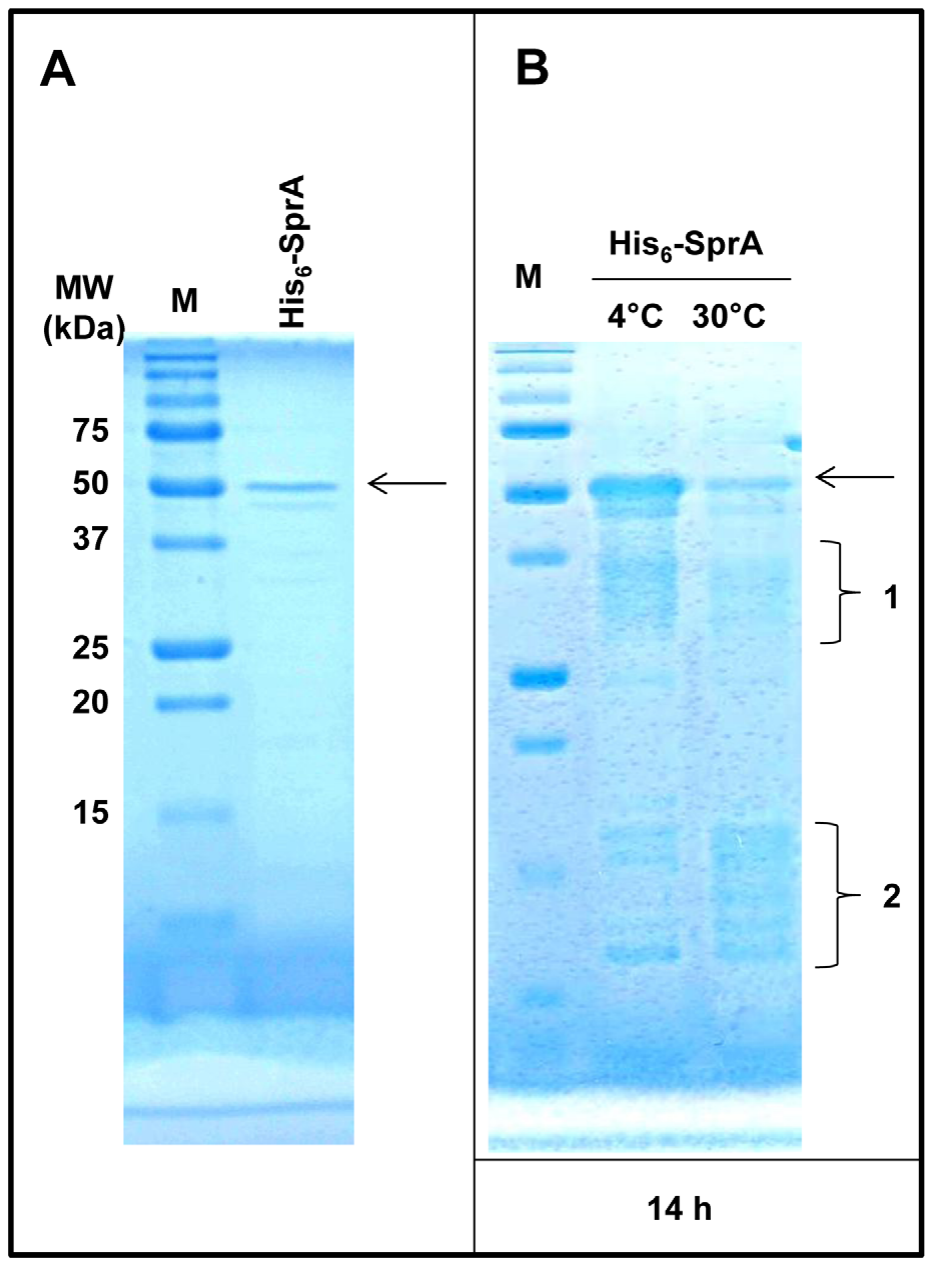
Purification and self-proteolysis of SprA. Purified His_6_-SprA (A) was incubated at 4°C or 30°C for 14 h as described in the Materials and Methods (B). The arrows indicate purified His_6_-SprA. The self-proteolysis of His_6_-SprA is shown in brackets 1 and 2 of B. M, molecular weight marker.

**Figure 5:**
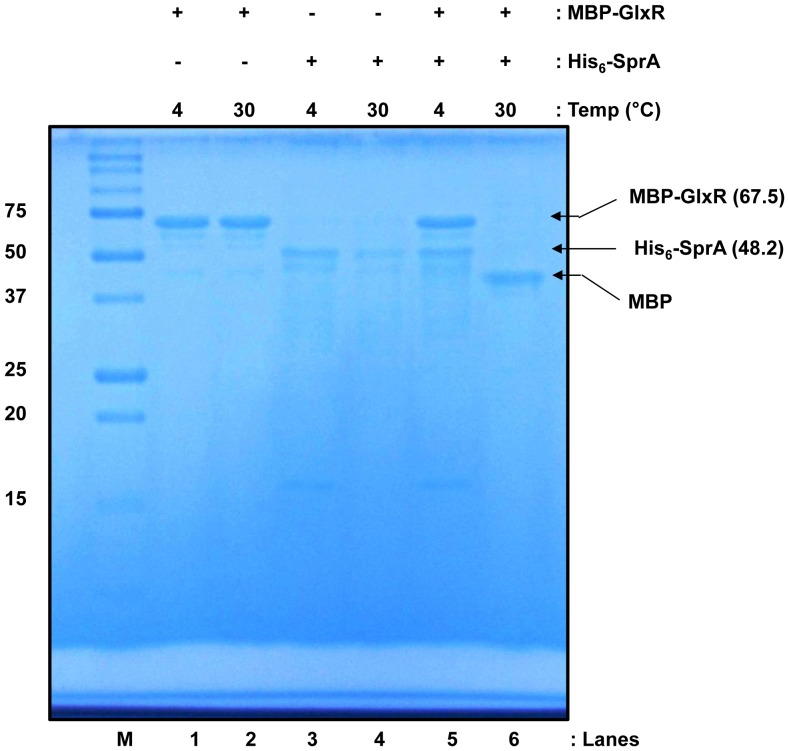
*In vitro* digestion of MBP-GlxR by His_6_-SprA. Protein purification and proteolytic assays were performed as described in the Materials and Methods. Proteins were incubated at 4°C or 30°C for 24 h, analyzed on a 15% SDS-PAGE gel, and then visualized by staining with Coomassie brilliant blue G250. Molecular weights are shown in kDa. MBP, maltose-binding protein; M, molecular weight markers; and OD, optical density.

**Figure 6:**
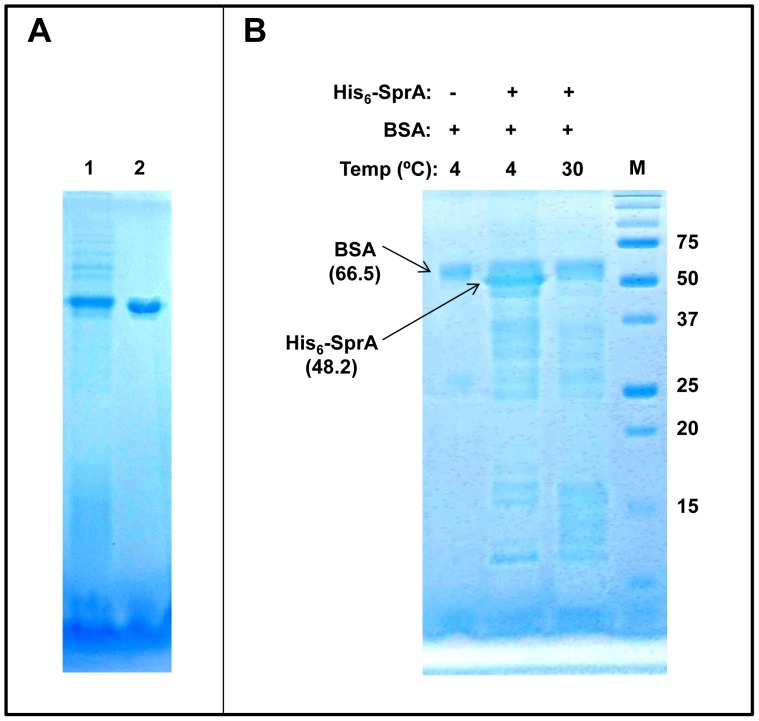
Proteolytic specificity of His_6_-SprA. Proteolytic assays were performed as described in the Materials and Methods. Proteins were incubated at 4°C or 30°C for 24 h, analyzed on a 15% SDS-PAGE gel, and then visualized by staining with Coomassie brilliant blue G250. Lanes 1 and 2 (A) show digested His_6_-SprA (identical to lane 6 in Figure 5) and purified MBP, respectively. (B) Digestion of bovine serum albumin (BSA) with His_6_-SprA. Molecular weights are shown in kDa, and M denotes the protein marker.

Because SprA was isolated based on its capability to interact with GlxR, we aimed to investigate protein-protein interactions in a purified system. The overexpressed MBP-GlxR fusion protein was soluble and could be purified using a conventional approach without difficulty. However, the overexpressed His_6_-SprA fusion protein was insoluble due to the formation of inclusion bodies. Therefore, we used a different purification approach, in which His_6_-SprA fusion protein was denatured with urea, bound to the column, and renatured on the column by slowly removing urea before elution. Once the protein was purified, it remained soluble (Figure 4A). However, during the purification process, partial degradation of His_6_-SprA was observed. Subsequently, we tested the stability of the purified His_6_-SprA by incubating it at 4°C or 30°C for 14 h. As shown in Figure 4B, the 48.2-kDa His_6_-SprA fusion protein underwent self-proteolysis during incubation at 4°C: SDS-PAGE revealed that fractions of purified SprA were digested to 27- to 35-kDa proteins (see Figure 4B, bracket 1). When the incubation was performed at 30°C, even smaller fragments were formed (see Figure 4B, bracket 2), indicating nearly complete digestion of His_6_-SprA. Although serine proteases are commonly synthesized as zymogens, which are converted to active enzymes via proteolytic cleavage at a particular peptide bond, no major identifiable protein band was detected in His_6_-SprA. In contrast, the 67.5-kDa MBP-GlxR fusion protein remained stable during purification and incubation; apparent self-proteolysis was not noted on performing SDS-PAGE (Figure 5, lanes 1 and 2) after a 24-h incubation period at 4°C or 30°C. After observing the proteolytic activity of His_6_-SprA, we tested it against MBP-GlxR. To minimize activity loss, we did not remove the protein fusion partners, such as MBP and His_6_, from each protein, eliminating additional purification steps. Interestingly, when the MBP-GlxR and His_6_-SprA fusion proteins were incubated together, the protein band corresponding to MBP-GlxR, as well as the band corresponding to SprA (Figure 5, lane 6), disappeared completely. However, a new 45-kDa protein band was detected by SDS-PAGE and remained stable. The M_r_ of the protein (Figure 6A, lane 1) was nearly identical to that of purified MBP (see Figure 6A, lane 2). To elucidate its nature, we excised the band from the gel for quadrupole time-of-flight (Q-TOF) analysis. The amino acid composition of the band indicated that the protein product was MBP, suggesting that SprA is specific for its GlxR target. We performed additional analyses by incubating His_6_-SprA with BSA. As shown in Figure 6B, proteolysis of BSA was not observed, whereas self-proteolysis of His_6_-SprA was evident. Although we observed digestion of GlxR by SprA, the reaction was slow, taking a maximum of 24 h. This result could be due to the poor activity of SprA, which was purified using an on-column refolding procedure. The digestion of GlxR by SprA was only observable with fresh protein preparations. In addition, the fusion partners MBP and His_6_ may have hindered protein-protein interaction. Nevertheless, these data indicate that, although unstable, SprA acts on the globular form of GlxR and digests it with specificity. Furthermore, self-proteolysis of SprA may indicate the importance of controlling the availability of the protein in cells for GlxR and other proteins.

### Effects of cAMP on the Proteolysis of GlxR by SprA

**Figure 7:**
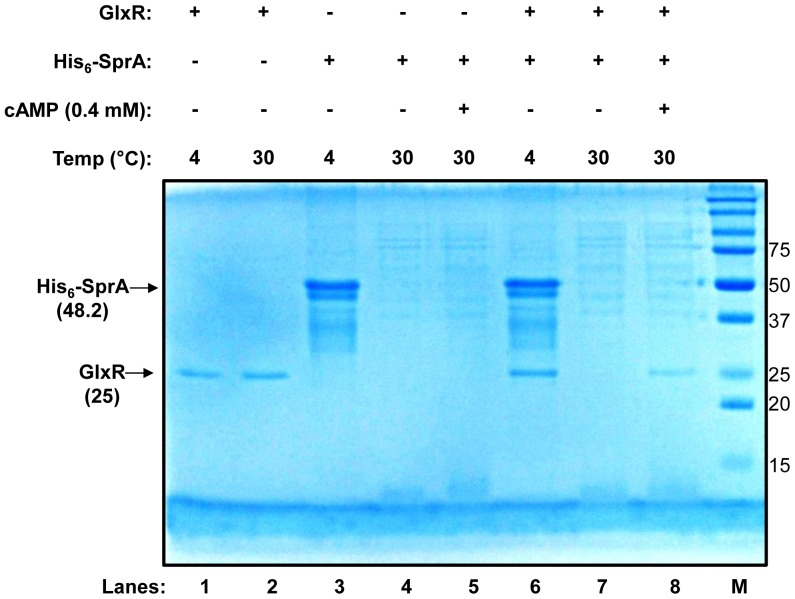
Effects of cAMP on *in vitro* digestion of GlxR by His_6_-SprA. Protein purification and proteolytic assays were performed as described in the Materials and Methods. Proteins were incubated at 4°C or 30°C for 20 h, analyzed on a 15% SDS-PAGE gel, and then visualized by staining with Coomassie brilliant blue G250. Molecular weights are shown in kDa. M, molecular weight markers; and MBP, maltose-binding protein.

Intracellular cAMP levels in *C. glutamicum* are elevated during growth on glucose, especially in the early log phase, and low during growth on acetate. The DNA-binding activity of GlxR is also modulated by cAMP. Once SprA was found to specifically proteolyze GlxR, the effects of cAMP on the proteolysis were investigated, using native GlxR by removing MBP from MBP-GlxR. As shown in Figure 7 (lanes 1 and 2), purified GlxR remained stable during the incubation period at 4°C or 30°C. When GlxR was incubated in the presence of SprA at 30°C (see Figure 7, lane 7), both proteins completely disappeared, indicating complete proteolytic digestion. However, when the reaction was performed in the presence of cAMP, fractions of GlxR survived proteolysis (Figure 7, lane 8). Complete self-proteolysis of SprA was still evident in the reaction (Figure 7, lanes 5 and 8). These data demonstrate that, although GlxR is a substrate for SprA, it can still be protected from proteolysis by SprA depending on the physiological conditions of the cell.

### Effects of *sprA* on the Expression of *aceA*, *aceB*, and *icd*

The genes *aceA* and *aceB*, which encode ICL and MS, respectively, are repressed by *glxR* in cells supplied with glucose as the sole carbon source. Therefore, one can speculate that *C. glutamicum* P_180_-*sprA* cells overexpressing the *sprA* gene may have depleted intracellular GlxR owing to proteolysis by SprA and may show derepression of *aceB* and *aceA* genes, even in cells supplied with glucose as the sole carbon source. Conversely, *C. glutamicum* cells grown on glucose have elevated levels of intracellular cAMP [6]. Therefore, as shown in Figure 7, SprA may be unable to proteolyze GlxR due to high intracellular cAMP levels in glucose-grown cells, resulting in the repression of *aceA* and *aceB*. Furthermore, the *C. glutamicum* Δ*glxR* strain, which has no intracellular GlxR, is known to have a severe growth defect [24], [25], whereas the *C. glutamicum* P_180_-*sprA* strain, which is considered equivalent to the Δ*glxR* strain, showed reduced but reasonable growth on glucose minimal medium (see Figure 3 A), suggesting that the intracellular GlxR might be intact. To determine whether the *in vitro* data of the present study agree with the reported observations for whole cells, we measured the ICL and MS activities from P_180_-*sprA* cells supplied with glucose as the carbon source. As shown in Table 2, the ICL and MS activities in P_180_-*sprA* cells were nearly comparable to those in the wild-type strain. These observations suggest that GlxR is almost intact in the cells, supporting the growth of the strain (see Figure 3A). Conversely, when acetate was supplied as the carbon source, as in the wild-type strain, derepression of glyoxylate bypass enzymes was observed in both the Δ*sprA* and P_180_-*sprA* strains (see Table 2). The observation that the level of derepression in the Δ*sprA* strain was comparable to that in the wild-type and P_180_-*sprA* strains may indicate that the amount of intracellular SprA is not important under those growth conditions. The results obtained with both Δ*sprA* and P_180_-*sprA* strains on glucose or acetate as the carbon source are in accordance with the *in vitro* data and suggest that the *in vivo* state of GlxR, the activity of which responds to cAMP, may determine its fate as the substrate for the SprA protein (see Discussion).

**Table 2:** Enzyme activities of isocitrate lyase (ICL), malate synthase (MS), and isocitrate dehydrogenase (ICDH) in cell extracts of *C. glutamicum* cells^a^.

			Specific activity, μmol min^-1^ mg^-1^		
Carbon source	Strains	Phenotype	ICL	MS	ICDH
Glucose	AS019E12	Wild type	0.116	0.092	0.008
	HL1385	Δ*sprA*	0.098	0.096	0.004
	HL1389	P_180_-*sprA*	0.110	0.098	0.003
Acetate	AS019E12	Wild type	0.428	0.506	0.008
	HL1385	Δ*sprA*	0.389	0.508	0.009
	HL1389	P_180_-*sprA*	0.391	0.434	0.008

a The enzymes were induced by growing *C. glutamicum* AS019E12 cells to the late log phase on MCGC minimal medium. Cells were harvested, disrupted, and assayed for the activity as described in the Materials and Methods. The activities represent one of three independent experiments.

Next, we measured the activity of isocitrate dehydrogenase (ICDH, encoded by *icd*), which is a key enzyme for the tricarboxylic acid cycle and converts isocitrate to α-ketoglutarate and CO_2_ with the concomitant release of reduced nicotinamide adenine dinucleotide phosphate. In *C. glutamicum*, the enzyme is constitutively formed independent of the growth substrate [41], [42]. Kim et al [6] have observed increased ICDH activity (twofold higher) in glucose-grown *C. glutamicum* cells harboring multiple copies of *glxR*, suggesting a positive role of *glxR* in *icd* expression, whereas no such effect was observed in acetate-grown cells. As shown in Table 2, the ICDH activity observed in the P_180_-*sprA* strain was only 32% of that of the wild-type strain, suggesting that the stimulatory effect of *glxR* on *icd* was abolished in the P_180_-*sprA* strain. In accordance with the finding by Kim et al [6], the enzyme activities of ICDH were found to be unaffected in acetate-grown cells regardless of the presence of the P_180_-*sprA* plasmid or Δ*sprA* mutation (see Table 2). The decrease in ICDH activity in glucose-grown cells was speculated to be due to the transcriptional control of *icd* because activity was unaffected in acetate-grown cells. Although we do not know the cause of this result, we also observed a decrease in ICDH activity in Δ*sprA* cells grown with glucose as the sole carbon source. The activity was 50% of that in the wild-type strain (see Table 2). Decreases in ICDH activity in glucose-grown Δ*sprA* cells suggest the involvement of additional regulatory proteins (see Discussion). We hypothesized that the decrease in ICDH activity in P_180_-*sprA* and Δ*sprA* mutant cells contributed to the slower growth of the cells on glucose minimal medium (see Figure 3A). In support of this hypothesis, growth differences in the cells grown on acetate minimal medium were marginal (see Figure 3B). Similarly, the attenuation of ICDH activity decreases biomass yield and increases lysine production in lysine-producing *C. glutamicum* cells [43]. Collectively, these data suggest that *sprA* has unknown regulatory roles and that its involvement in *glxR*-mediated regulation may be more complex than anticipated.

### Identification of Proteins under the Control of *sprA*

**Figure 8:**
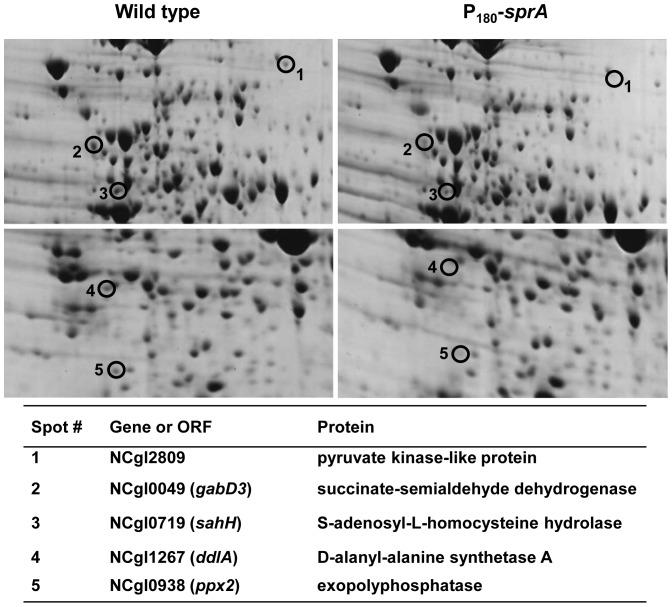
Identification of protein affected in *sprA*-overexpressing *C. glutamicum* (P_180_-*sprA*). 2D-PAGE was performed as described in the Materials and Methods. A total of 150 μg of protein was loaded onto each gel. The identity of each protein spot was determined by electrospray ionization mass spectroscopy.

**Figure 9:**
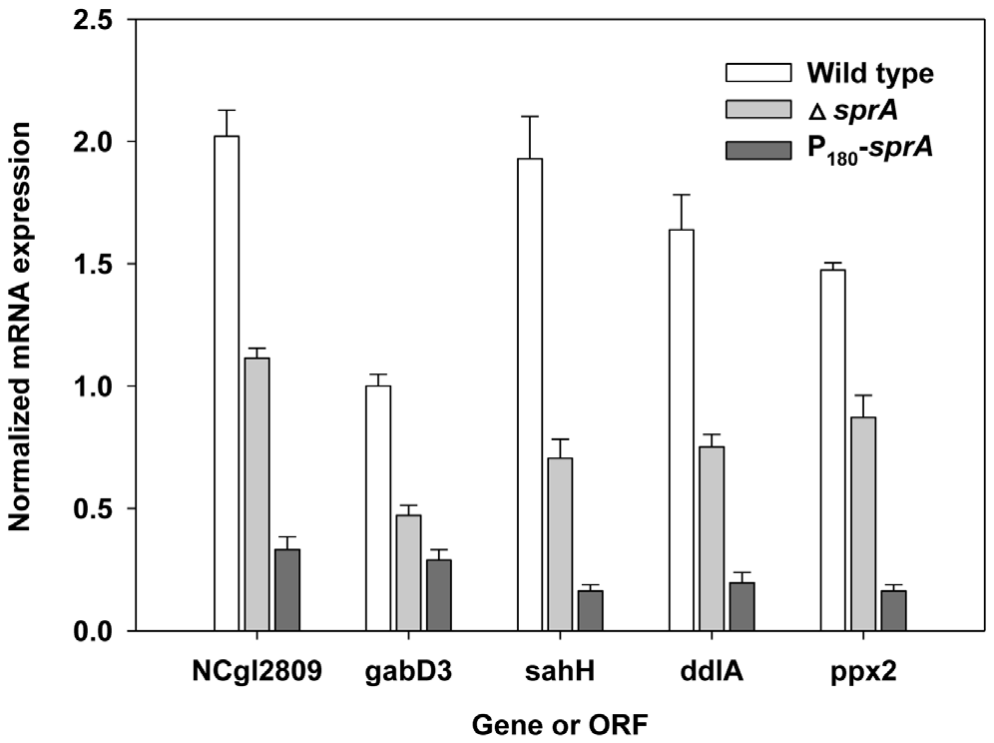
Transcription of genes in *C. glutamicum* strains. Cells were grown in glucose MCGC media, and mRNA levels were measured by RT-qPCR as described in the Materials and Methods. The data represent three independent experiments. Bars indicate mRNA levels in wild-type (empty bars), Δ*sprA* (light grey bars), and P_180_-*sprA* (dark grey bars) strains.

After investigating the role of *sprA* in general cell physiology (growth differences are shown in Figure 3, and differences in ICDH activity between P_180_-*sprA* and Δ*sprA* strains are in Table 2), we assumed that additional proteins might be affected in P_180_-*sprA* cells. To identify proteins with altered expression, we used 2D-PAGE analysis and compared the protein profiles of P_180_-*sprA* cells with those of wild-type cells. We chose P_180_-*sprA* cells because they exhibited a more severe growth defect than Δ*sprA* cells. Our analysis revealed the following five protein spots (Figure 8): pyruvate kinase-like protein (NCgl2809), succinate-semialdehyde dehydrogenase (NCgl0049), *S*-adenosyl-l-homocysteine hydrolase (NCgl0719), d-alanyl-alanine synthetase A (NCgl1267), and exopolyphosphatase (NCgl0938). These proteins showed decreased spot density in the P_180_-*sprA* strain. Although we found a pyruvate kinase-like protein, pyruvate kinase is a well-known enzyme that catalyzes the last step in glycolysis, forming ATP from ADP by converting phosphoenolpyruvate to pyruvate [44]. Succinate-semialdehyde dehydrogenase activity has been suggested to play important roles in nitrogen metabolism in *E. coli* [45]. *S*-Adenosyl-l-homocysteine hydrolase is an important regulator of *S*-adenosylmethionine-dependent biological methylation reactions and catalyzes the reversible breakdown of *S*-adenosyl-l-homocysteine to adenosine and homocysteine [46]. d-Alanyl-d-alanine ligase (or synthetase) is a cytoplasmic enzyme that provides the d-alanyl-d-alanine dipeptide substrate in the initial cytoplasmic phase of peptidoglycan biosynthesis [47]. Exopolyphosphatase catalyzes the hydrolysis of exopolyphosphate, which plays a role in cell stress, starvation, survival, and virulence [48]. Next, to determine whether the decreased intensity in protein spot was due to a decrease in the transcription of the corresponding genes or increased proteolysis by the SprA, we measured the amount of corresponding mRNA using RT-qPCR. The amount of mRNA in P_180_-*sprA* cells decreased to approximately 10-30% of that in the wild-type strain (Figure 9), showing that the decrease in spot intensity was caused by decreased transcription of the corresponding genes. Reduced transcription of the genes was also observed in Δ*sprA* cells (Figure 9), suggesting that the action of SprA was conveyed via transcriptional regulators, such as GlxR. Although genes in the GlxR regulon are mostly known from studies involving *in silico* genome analysis and *in vivo* and *in vitro* DNA-binding assays [20], [21], [23], the genes that we identified in the above analysis have not been listed among those in the GlxR regulon. However, the known functions of these proteins, such as those in energy and carbon metabolism (NCgl2809), nitrogen metabolism (NCgl0049), methylation reactions (NCgl0719), peptidoglycan biosynthesis (NCgl1267), as well as stress, starvation, and survival (NCgl0938) are associated with the functional category in which *glxR* plays a role. All of the data obtained suggest that SprA performs an important role not only in *glxR*-mediated gene regulation but also in other areas of cell physiology.

## Discussion

In this study, we used a two-hybrid system to isolate the subtilisin-like serine protease SprA as a partner that interacts with GlxR. In the two-hybrid system, the bait (GlxR) and target (SprA) proteins are expressed as fusion proteins linked to the λcI repressor and RNA polymerase α subunit, respectively. Interactions between the bait and target proteins stabilizes the interaction of RNA polymerase with its weak promoter, resulting in the expression of *aadA* and *his3* reporter genes, which confer Str+ and His+, respectively. The *in vivo* GlxR-SprA interaction was quantified using RT-qPCR, which measured *his3* transcription, and determined to be low relative to that of the positive control. This decrease could be due to the proteolysis of GlxR with the interacting SprA, decreasing the transcription of the reporter genes. This assumption is highly supported by the observed GlxR proteolysis by SprA in a purified system. Self-proteolysis of SprA may have also contributed to the decrease in the reporter gene transcription. In addition, the binding of GlxR to SprA as a proteolytic substrate may not be strong enough to stabilize interactions between RNA polymerase and its promoter to allow transcription.

The finding that GlxR interacts with a protease is surprising because the common role of proteases is protein quality control, such as the degradation of damaged or unfolded proteins [49]. Subtilisin-like serine proteases are also generally secreted extracellularly to scavenge nutrients, although intracellular roles are also commonly found. Proteases are known to conduct critical regulatory functions via the proteolysis of regulators, enzymes, and other proteins [26], [50]. Regulators that show either functional or structural similarity or both to GlxR also exist. For example, the global transcription factor FNR, which plays a role in anaerobiosis in *E. coli*, is controlled by proteolysis [51], [52] as well as the oxidative stress response regulator Spx in low-GC Gram-positive bacteria [53]. The global regulator Mlc, which is a transcriptional repressor of sugar-metabolizing enzymes and uptake systems in *E. coli*, is also regulated by proteolysis [54]. A protease system is also involved in the cellular turnover of FixK2, which is a CRP-like transcription factor that controls the endosymbiotic lifestyle of *Bradyrhizobium japonicum* [55]. In *C. glutamicum*, the Clp complex and FtsH protease are involved in nitrogen control through action on GlnK [56], [57]. In addition, *C. glutamicum* FtsH protease has been implicated in the regulation of energy and carbon metabolism as well as in amino acid biosynthesis [58]. Therefore, it is not surprising to find SprA proteolyzing the global regulator GlxR with specificity. A serine protease MycP1 from *M. tuberculosis*, a close relative of *C. glutamicum*, also performs a novel role with defined substrate specificity [59]. Considering the constitutive transcription of *glxR* (see Figure 2), one can speculate the involvement of additional regulatory mechanisms, such as proteolysis, for GlxR.

In general, serine proteases show broad substrate specificity. Although many function as general proteases for unfolded substrates, some cleave only one substrate. In addition, many prokaryotic serine proteases show domain architectural diversity and thus function in an organism-specific manner [38]. Several observations suggest that *sprA* performs a novel role in *C. glutamicum*: (a) Both deletion and overexpression of the gene resulted in a retarded growth phenotype, indicating a global role for the gene. (b) The transcription of *sprA* showed an atypical temporal pattern, suggesting a specialized role for the gene. Typically, most subtilisin genes are expressed at low levels during the log growth phase [60]. *M. tuberculosis myc* genes are also expressed constitutively [39]. (c) SprA was not homologous to other subtilisin-like serine proteases. (d) Complete self-proteolysis of SprA is unique and suggests that tight control of SprA availability is important owing to its regulatory role in cell physiology. (e) Although five *myc* genes are present in closely related *M. tuberculosis*, only one homologous gene (*sprA*) is found in *C. glutamicum*, suggesting a novel role.

Although we initially isolated SprA as a protein that specifically interacted with GlxR, its regulatory role now appears to be much more complex than we expected. First, the P_180_-*sprA* strain showed no lethal phenotype, indicating that GlxR was still active in the cell but reduced cell growth, which suggests that other cellular functions were affected. In addition, the proteins identified with 2D-PAGE are not known to be included in the GlxR regulon, suggesting that putative GlxR-binding motifs are absent in the regulatory region of the identified genes. Furthermore, the activity of ICDH was severely affected in Δ*sprA* and P_180_-*sprA* cells. *Icd*, encoding ICDH, showed no *glxR*-binding motif in its promoter and regulatory regions and is excluded from the GlxR regulon. However, this gene is still regulated by *glxR*, as noted in our study and by Kim et al [6], suggesting unknown regulation in central carbon metabolism. In *E. coli*, the *icd* gene has two different promoters and is controlled by a complex regulatory system involving the global regulatory proteins ArcA, Fnr, and Cra [61], [62]. The expression of *icd* in *E. coli* is controlled by carbon and oxygen availability; its expression decreased during anaerobic growth and increased in the presence of poor carbon sources, such as acetate. In this type of control, ArcA and Fnr act negatively, and Cra exerts positive control. Analogous to this, our results suggest a complex regulatory mechanism for the *C. glutamicum icd* gene as well.

The activity of GlxR is modulated by cAMP. In all the cases studied, the *in vitro* binding of GlxR to its target binding sites is strictly dependent on the presence of cAMP. Unlike the levels in *E. coli*, intracellular cAMP levels in *C. glutamicum* are reported to be elevated during growth on glucose and low during growth on acetate [6]. Therefore, cells grown on glucose may have GlxR in its holo form; that is, GlxR may exist as a cAMP-bound protein. However, as shown in Figure 7, SprA may not act on the holo form of GlxR. We observed proteolysis of GlxR by SprA only in the absence of cAMP, suggesting that only the apo form of GlxR could serve as the substrate for SprA. The *in vitro* binding of GlxR to its target DNA requires the addition of cAMP to the assay mixture, probably to convert the GlxR into its cAMP-bound holo form, which is considered active and protected from proteolysis by SprA, thereby performing its function by repressing glyoxylate bypass genes. As a result, ICL and MS activities are minimal in glucose-grown Δ*sprA* and P_180_-*sprA* strains, as shown in Table 2. The reasonable growth of the P_180_-*sprA* strain on glucose minimal medium (see Figure 3A) suggests that intracellular GlxR is mostly intact and supports the above-mentioned hypothesis. Conversely, in acetate-grown cells, GlxR probably exists in its apo form; that is, it does not bind to cAMP owing to low intracellular cAMP levels. If cAMP is absent in the cells, the DNA-binding activity of GlxR remains minimal, thereby derepressing the glyoxylate bypass genes. Under this condition, the overexpression of *sprA* (P_180_-*sprA*) or deletion of the gene (Δ*sprA*) has no significant effect on the expression of glyoxylate bypass genes, as shown in Table 2. In wild-type cells, residual intracellular GlxR in its apo form can be further proteolyzed by SprA and cleared from the cell, preventing residual GlxR from performing any regulatory role (see below). Thus, our hypothesis that only the apo form of GlxR, which is not bound to cAMP, serves as the substrate for SprA is logical, because otherwise, uncontrolled degradation of GlxR by SprA would occur and disturb the cell physiology by depleting GlxR.

We can also speculate about why proteolysis is used to regulate GlxR, which has DNA-binding activity that is already controlled by intracellular cAMP levels. Any protein subject to regulated synthesis will also be degraded as part of a regulatory network [26], and this arrangement may be particularly important for proteins, such as *glxR*, that show autoregulation [19] because the basal levels of the protein can be kept even lower if the protein is subject to degradation. In the case of global regulators, small changes in synthesis may inappropriately switch on genes and disturb cell physiology. One of the roles of SprA may be to keep the intracellular levels of GlxR below working concentrations, which may be why GlxR is the target for a serine protease rather than an ATP-dependent AAA+ protease that requires a more complex regulatory mechanism. Alternatively, the removal of GlxR may be important when it is no longer needed. GlxR is well expressed during growth (see Figure 2), but as conditions fluctuate, GlxR, which once served a useful function, likely requires clearance from the cell at times. The primary goal of SprA may be to remove GlxR when it is no longer needed. As mentioned above, cAMP probably plays a key role in GlxR-SprA interaction and the stimulation of proteolysis. The constitutive expression of *glxR*, temporal expression of the *sprA* gene (see Figure 2), and self-proteolysis of SprA to remove itself after use (see Figure 4) support this hypothesis.

In conclusion, we isolated SprA as an interacting partner for GlxR. *SprA* preferentially functions in the log phase and mediates the proteolysis of GlxR with specificity. In addition, *sprA* appears to play additional regulatory roles in general cell physiology. The self-proteolytic activity of SprA suggests that the immediate and timely removal of SprA is also important to ensure precise regulatory control.
